# Evaluation of a Simulation Based Training Course for Non-Consultant Hospital Doctors (NCHDS) in Psychiatry

**DOI:** 10.1192/j.eurpsy.2024.1665

**Published:** 2024-08-27

**Authors:** K. Corrigan, G. Crudden, A. Doherty

**Affiliations:** ^1^ St Vincent’s University Hospital; ^2^ St. Vincent’s University Hospital; ^3^Holles Street, The National Maternity Hospital; ^4^The Mater Misericordiae Hospital, Dublin, Ireland

## Abstract

**Introduction:**

Simulation-based training (Sim) is an established method of teaching in medical education and can help bridge the gap between medical theory and clinical practice. While sim is well-established in medical and surgical specialties, it is less well developed in psychiatry. Psychiatric emergencies often occur out of hours when there are fewer senior staff available on-site. Sim offers a safe setting for development of essential clinical skills with carefully delivered feedback.

Sim can be high-cost involving specialized simulation facilities, especially when utilising high-fidelity equipment. Even lower-fidelity techniques requiring standardized patients (SPs) require funding for actors and this can be a barrier to utilising Sim.

**Objectives:**

We piloted a Sim course to NCHDs working in psychiatry in a tertiary university hospital with the aim of improving trainee skills and confidence in managing psychiatric emergencies on-call including risk assessment, involuntary admission and acute behavioural disturbance. A low-fidelity approach was taken with minimal use of SPs.

**Methods:**

A sim handbook developed by Irish Centre for Applied Patient Safety and Simulation (ICAPSS) was used for reference in developing the simulation modules. Three modules were delivered in a structured manner over three hours; involuntary admission, risk assessment and management of acute behavioural disturbance. Each module involved the simulation exercise (20 minutes) followed by debrief (20 minutes). The facilitated debrief involved open discussion and prompted reflective learning. Anonymous, paper-based questionnaires were used to collect feedback on participants’ experience of the training.

**Results:**

There were 12 attendees and ten participants completed the feedback. All participants (100%, n=10) agreed or strongly agreed that sim helped them to learn and all agreed that the topics covered were relevant to their clinical role. All participants (100%, n=10), indicated that they enjoyed the workshop. Eighty percent (n=8) agreed or strongly agreed that they would like to do more sim-based workshops. The supportive environment and debrief sessions were reported as the most enjoyable aspects of the workshop.

**Conclusions:**

Participants unanimously agreed that the training was useful to them in their clinical roles and helped them to learn. Sim was effective in teaching high risk complex psychiatric cases to psychiatry NCHDs and consideration should be given to expand this teaching method within postgraduate psychiatry training in Ireland.

**Disclosure of Interest:**

None Declared

